# Interpreting Whole-Genome Sequence Analyses of Foodborne Bacteria for Regulatory Applications and Outbreak Investigations

**DOI:** 10.3389/fmicb.2018.01482

**Published:** 2018-07-10

**Authors:** Arthur W. Pightling, James B. Pettengill, Yan Luo, Joseph D. Baugher, Hugh Rand, Errol Strain

**Affiliations:** Biostatistics and Bioinformatics, Center for Food Safety and Applied Nutrition, U.S. Food and Drug Administration, College Park, MD, United States

**Keywords:** whole-genome sequence, genomic epidemiology, outbreak investigation, interpretation, phylogenetics, *Listeria monocytogenes*, *Salmonella enterica*, *Escherichia coli*

## Abstract

Whole-genome sequence (WGS) analysis has revolutionized the food safety industry by enabling high-resolution typing of foodborne bacteria. Higher resolving power allows investigators to identify origins of contamination during illness outbreaks and regulatory activities quickly and accurately. Government agencies and industry stakeholders worldwide are now analyzing WGS data routinely. Although researchers have published many studies that assess the efficacy of WGS data analysis for source attribution, guidance for interpreting WGS analyses is lacking. Here, we provide the framework for interpreting WGS analyses used by the Food and Drug Administration’s Center for Food Safety and Applied Nutrition (CFSAN). We based this framework on the experiences of CFSAN investigators, collaborations and interactions with government and industry partners, and evaluation of the published literature. A fundamental question for investigators is whether two or more bacteria arose from the same source of contamination. Analysts often count the numbers of nucleotide differences [single-nucleotide polymorphisms (SNPs)] between two or more genome sequences to measure genetic distances. However, using SNP thresholds alone to assess whether bacteria originated from the same source can be misleading. Bacteria that are isolated from food, environmental, or clinical samples are representatives of bacterial populations. These populations are subject to evolutionary forces that can change genome sequences. Therefore, interpreting WGS analyses of foodborne bacteria requires a more sophisticated approach. Here, we present a framework for interpreting WGS analyses that combines SNP counts with phylogenetic tree topologies and bootstrap support. We also clarify the roles of WGS, epidemiological, traceback, and other evidence in forming the conclusions of investigations. Finally, we present examples that illustrate the application of this framework to real-world situations.

## Introduction

Foodborne illness is a substantial hazard to human health and a significant burden on the economy. Economists have estimated the monetary cost of medical care and lost productivity due to consumption of contaminated food to be over $15 billion annually (Hoffman et al., 2015). In addition, costs to industry stakeholders due to product recalls are significant. Individual firms have reported losses of up to $100 million, while losses to sectors have topped $1 billion (Grocery Manufacturers Association, 2011). Thus, it is imperative that government entities and industry stakeholders eliminate contaminated products from the food supply as quickly as possible to reduce both the human and monetary costs of foodborne illnesses.

Determining sources of foodborne illnesses requires subtyping methods that accurately identify bacterial pathogens (Farber, 1996; Allard et al., 2012). The ability to quickly identify and track foodborne bacterial pathogens is critical to lessen the adverse impact on human health and to reduce the numbers of contaminated products that may need to be recalled. Whole-genome sequence (WGS) analysis enables rapid identification of outbreaks and allows investigators to resolve them when they are smaller, resulting in fewer illnesses (den Bakker et al., 2014; Jackson et al., 2016). WGS analysis provides higher resolving power than subtyping methods that recently dominated the food safety landscape, such as pulsed-field gel electrophoresis and serotyping (Pightling et al., 2015). Increased resolution allows investigators to more confidently identify bacteria that are members of a contamination event or outbreak and exclude those that are not (den Bakker et al., 2014; Jackson et al., 2016). The ability to focus regulatory activities and foodborne outbreak responses quickly enables investigators to use resources efficiently and to protect the public health, while minimizing the impact on commerce.

The most extensive and best-known application of WGS to food safety is the GenomeTrakr Network (GT^1^). GT is an international collaboration of government and academic laboratories that includes the U.S. Food and Drug Administration (FDA), Centers for Disease Control and Prevention, U.S. Department of Agriculture, U.S. National Center for Biotechnology Information (NCBI), state health departments, and international partners (Allard et al., 2016). Member laboratories have sequenced vast numbers of *Listeria monocytogenes, Salmonella enterica*, and *Escherichia coli* genomes (Stevens et al., 2017). In addition, non-member laboratories have submitted sequencing data to the GT database. Bacteria isolated from food, environmental, and clinical samples are included, providing a powerful means of identifying potential sources of illnesses. The data are publically available at the NCBI in GT associated BioProjects. As of February 21, 2018, network laboratories have submitted sequence data for 16,864 *L. monocytogenes*; 105,330 *S. enterica*; and 38,347 *E. coli* and *Shigella* ^2^. The NCBI also provides tools for exploring and analyzing the data ^3^. The availability of the database and preliminary analyses promotes transparency and supports the WGS community in developing WGS-based approaches to ensuring a safe food supply (Allard et al., 2016).

An additional benefit of the GT database is that it enables objectivity in the analysis of WGS data. Analyses at the FDA begin by comparing genome sequences to the database, with no assumptions regarding taxonomic relationships. Subsequent WGS analyses include all closely related genomes, with no editing or removal of taxa. In the final interpretation of WGS reports, investigators evaluate all relationships between a query and closely related genomes. This approach supports unbiased WGS analyses.

The FDA uses WGS data to assess levels of genetic relatedness among isolates. The presence of tens of SNPs indicates that bacteria are genetically similar and recently originated from the same source. By contrast, when isolates are distant in time or geographic origin, there are hundreds, thousands, or tens of thousands of SNPs between their genome sequences, indicating that they did not recently arise from the same source. The similarity of isolates also allows investigators to assess whether bacterial strains in production environments are “resident” strains (i.e., bacterial populations that have persisted in facilities or supply chains for months or years) (Ferreira et al., 2014; Stasiewicz et al., 2015). The status of foodborne pathogens as resident is important for enforcement actions as it provides insight into the effectiveness of a firm’s safety management plan. In addition, WGS results, along with the locations of positive samples within facilities, can assist stakeholders in locating and eradicating foodborne pathogens. This approach was used successfully to identify transmission routes for bacteria in a hospital setting (Snitkin et al., 2012). WGS analysis is a powerful tool for enforcement activities that provides specific and actionable information that empowers stakeholders to supply safe and sanitary food products.

The globalization of food supply chains presents challenges to food safety (Kruse, 1999; Keener et al., 2014). Foodborne pathogens can easily traverse jurisdictions (Imanishi et al., 2014; Moura et al., 2016; Self et al., 2016). This can be problematic when different agencies use incongruent procedures and workflows and do not arrive at identical conclusions. To address this issue, efforts to harmonize sequence data quality by the Global Microbial Identifier initiative are ongoing (Moran-Gilad et al., 2015). Similarly, the Genomic Epidemiology Ontology project seeks a global standard for the contextual information (metadata) that is associated with WGS data (Griffiths et al., 2017). A clear need exists for a global harmonization of interpretation guidelines to improve information sharing and to ensure the efficient use of WGS analyses during multi-jurisdictional events.

Currently, there is limited guidance available on interpreting the results of WGS analysis for source attribution studies. Although, guidance on interpreting WGS analysis for the detection of antimicrobial resistance genes is published (Aquilina et al., 2018). To foster debate, promote transparency, and advocate for general acceptance of interpretation methodology, the regulatory WGS community needs to explicitly state the considerations that analysts take into account when reaching conclusions. Here, we state our current framework for interpreting WGS analyses and present examples of its use. These examples highlight the challenges encountered while interpreting WGS analyses and the thought processes that drive solutions. We also emphasize how increased resolution contributes to better decision-making and more confident source attribution. Although WGS analysis is the primary focus of this paper, it is just one informative unit in the decision-making process during an outbreak response or regulatory activity. Epidemiology considerations, traceback information, and other data are critical to determining the most appropriate regulatory responses. This activity reduces the extent of outbreaks of disease and ensures that any regulatory actions are accurate, necessary, and promote the well being of both the public health and the food processing industry.

## Whole-Genome Sequence Data Generation and Bioinformatic Analysis

Laboratories produce whole-genome sequencing datasets from food, environmental, and clinical isolates with next-generation sequencing platforms (e.g., Illumina, Pacific Biosciences, and Oxford Nanopore technologies) and submit them to NCBI’s Sequence Read Archive under an appropriate GenomeTrakr BioProject ^4^. WGS datasets are analyzed at the NCBI with an automated pipeline that aggregates the isolates into clusters based on genetic differences ^5^. These differences are visualized with phylogenetic trees that can be used to identify closely related isolates. The FDA measures the numbers of SNPs between genomes of closely related isolates with the CFSAN SNP pipeline (Davis et al., 2015) and generates phylogenetic trees with the resulting SNP matrices. We calculate phylogenies from SNP matrices using a maximum likelihood approach to search for the best tree (identified from 10 runs) with GARLI v2.01.1067 (Zwicki, 2006), using the K80 and HKY nucleotide substitution models. To provide a measure of statistical support for the relationships inferred we generate bootstrap values from 1000 replicates.

## Defining Matches Between Bacterial Genome Sequences

**FIGURE 1:**
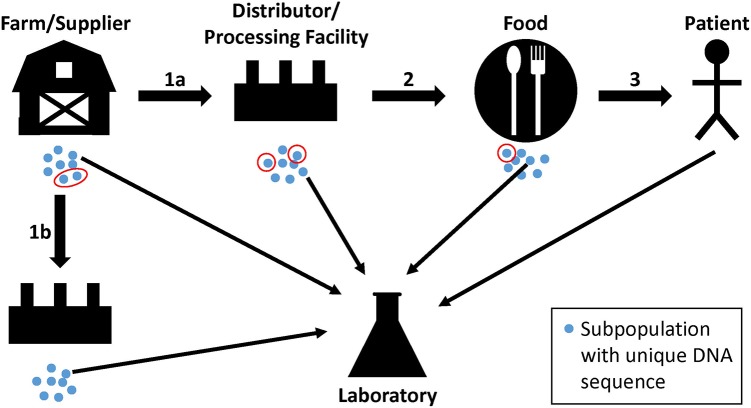
Hypothetical pathway of bacterial pathogens along the farm-to-fork continuum. Genetically unique subpopulations of bacterial pathogens (blue dots) can grow in farms or other suppliers from reservoir populations. Representatives of one or more subpopulations can be transmitted to distributors and processing facilities, food, and to patients (red circles; steps 1a, 2, and 3). At each step, the founding bacteria may grow into multiple subpopulations. Product, environmental, or clinical samples may yield representative bacteria from closely related subpopulations that are analyzed by the food safety laboratory (thin arrows). Farms and suppliers may have more than one customer, resulting in the dissemination of genetically identical bacteria to multiple distributors or food processing facilities (step 1b).

Investigators use WGS analyses to supplement traceback and epidemiological evidence during regulatory activities and outbreak investigations that identify sources of contamination. These sources are populations of bacterial pathogens that are often present on raw commodities and in the production lines of food processing facilities. The pathways that bacterial pathogens take from farm to fork can be complex, and bacteria can undergo evolutionary changes, especially over long periods of time (Spratt, 2004). Bacteria from a farm or other supplier can grow into genetically distinct subpopulations (**Figure 1**, blue dots). Members of one or more subpopulations (**Figure 1**, red circles) can be transmitted to a distributor or processing facility (**Figure 1**, step 1a), followed by further growth and division into additional subpopulations. Again, members of one or more of the subpopulations may be transmitted to food and, sometimes, a patient (**Figure 1**, steps 2 and 3). Importantly, sampling may not retrieve a representative from the same subpopulation that traveled from one point to another, but it may yield a representative of a closely related subpopulation (**Figure 1**, thin arrows). Also, a farm or other supplier may have additional customers, and genetically identical bacteria can be transmitted to multiple distributors or food processing facilities (**Figure 1**, steps 1a and 1b). We do not imply with the example that farms or other suppliers are the ultimate sources of bacterial pathogens; contamination can occur along any point in the continuum. Bacteria with non-identical genome sequences can originate from the same source, and identical genome sequences should not lead to presumptions of causality. The goal of investigators is to consider these complexities, along with epidemiological and traceback evidence, to determine whether two or more isolates arose from the same reservoir population.

A common measure of genetic relatedness that is used for outbreak investigations is the numbers of nucleotide differences (SNPs) between isolates. If there are a few (tens of) SNPs, the isolates are closely related, increasing the likelihood that they arose from the same source. The presence of many (hundreds or more) SNPs indicates that isolates are distantly related, implying that they did not originate from the same reservoir population. This observation is consistent with published studies we reviewed (**Table 1**). For each of these studies, SNPs were measured among isolates that were confirmed with epidemiology and traceback evidence to have originated from the same source. In addition, SNP thresholds were not used to define outbreak clusters. These studies did not rely upon the framework presented here but influenced its development. For this reason, we expect that it is compatible with most, if not all, interpretation guidelines used in the regulatory WGS community.

**Table 1:** Maximum pairwise SNPs measured during investigations into foodborne illness outbreaks and contamination events.

Organism	Maximum SNP count (number)	Maximum SNP count (range)			Reference
		<21	21–100	>100	
*E. coli*	4	X			Underwood et al., 2013
*E. coli*	15	X			Eppinger et al., 2011
*L. monocytogenes*	9	X			Chen et al., 2017c
*L. monocytogenes*	12	X			Chen et al., 2017a
*L. monocytogenes*	18	X			Li et al., 2017
*L. monocytogenes*	20	X			Wang et al., 2015
*L. monocytogenes*	21		X		Nielsen et al., 2017
*L. monocytogenes*	28		X		Gilmour et al., 2010
*L. monocytogenes*	29		X		Chen et al., 2017b
*L. monocytogenes*	42		X		Chen et al., 2016
*L. monocytogenes*	67		X		Jackson et al., 2016
*S. enterica*	2	X			Wuyts et al., 2015
*S. enterica*	3	X			Allard et al., 2016
*S. enterica*	3	X			Taylor et al., 2015
*S. enterica*	6	X			Hoffmann et al., 2016
*S. enterica*	12	X			Octavia et al., 2015
*S. enterica*	30		X		Leekitcharoenphon et al., 2014

*The maximum SNP counts for isolates that were traced back to the same source in the original study are presented. Whether the maximum SNP counts are less than 21 SNPs, 21 to 100 SNP, or greater than 100 SNPs is also indicated.*

It is important to note that different populations of bacteria contain different levels of genetic diversity that investigators must consider when interpreting WGS results. Evolutionary forces (e.g., genetic drift, natural selection, founder effects, and population bottlenecks) that result in SNPs drive this diversity. Also, isolation and culture conditions (such as multiple passages) can result in the accumulation of small numbers of additional SNPs (Allard et al., 2012). Therefore, we do not expect genome sequences arising from the same source of contamination to be identical. Investigators should not take nucleotide differences between genomes derived from the same source as indicators of uncertainty. Rather, these differences are manifestations of events (many of them unknowable) that occurred during the evolutionary histories of bacterial populations. This lack of knowledge about the evolutionary histories of bacterial populations does not diminish the conclusions arrived at through logical interpretation of WGS analyses (Wilson et al., 2013). It is also important to keep in mind that investigators do not usually have prior knowledge regarding the locations of bacterial populations within facilities. Inspectors can only collect representatives with a sampling regime that reflects their best judgment. It is a near certainty that WGS analyses of environmental isolates underestimate the true genetic diversity of bacterial populations.

**FIGURE 2:**
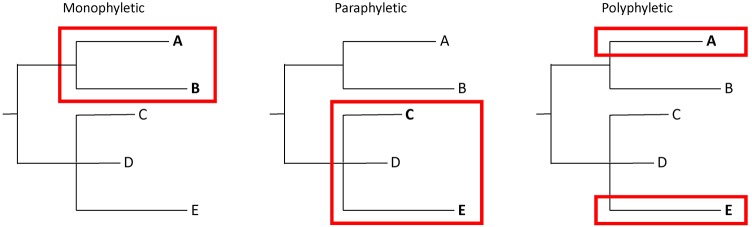
Illustration of monophyletic, paraphyletic, and polyphyletic groupings. A monophyletic topology exists when isolates of interest (e.g., A and B) group together to the exclusion of all others. A paraphyletic topology is one in which isolates of interest (e.g., C and E) group together but not to the exclusion of all others (e.g., D). A polyphyletic topology exists when isolates of interest do not form a group (e.g., A and E).

Bacteria from the same source may have significant numbers of SNPs. Thus, determining whether isolates match is not as straightforward as applying SNP thresholds. It is essential to put genetic distances into context with phylogenetic analyses that illustrate evolutionary relationships. This extra information requires a more nuanced interpretation of WGS analyses, in which tree topologies are considered in combination with SNP distances. An evaluation of whether isolates of interest are monophyletic (i.e., they group to the exclusion of all other isolates), polyphyletic (i.e., they appear in multiple parts of a tree), or paraphyletic (i.e., they group but not to the exclusion of all other isolates) is important when interpreting the results of WGS analyses (**Figure 2**). Confidence levels for relationships depicted by phylogenetic analyses should be assessed with bootstrap replicates (Felsenstein, 1985). Finally, epidemiological and traceback information must be considered. Appropriate interpretations of WGS analyses are only possible by integrating SNP distances, tree topology, bootstrap support, epidemiological data, and traceback data.

Laboratories routinely employ minimum sequence quality requirements and follow best practices for developing and using analytic pipelines (Lambert et al., 2017). However, whole-genome sequencing technologies and analytic workflows have non-zero error rates (Allard et al., 2012). Errors may yield misestimates of the numbers of SNPs between genomes (Pightling et al., 2014). However, they are unlikely to result in substantial changes to tree topologies (Allard et al., 2012). The match criteria presented here are designed to decrease the influences of errors on interpretation.

We present the general conditions for determining when two isolates arose from the same source in **Table 2**. We developed these conditions by studying published literature (**Table 1**) and by building a consensus within the FDA and larger WGS community on the use of WGS data during source attribution studies of foodborne bacterial pathogens. Our parameters for each type of evidence indicate whether they: (i) support, (ii) are neutral, or (iii) do not support the conclusion that isolates originated from the same population. Within this framework, there is strong support for a match when there are 20 or fewer SNPs and the phylogenetic analysis shows a monophyletic relationship with bootstrap support of 0.90 or higher. In contrast, 100 or more SNPs, polyphyly, and bootstrap support lower than 0.80 do not support a match. Isolates with any combination of evidence may ultimately be determined to match. However, epidemiological and traceback evidence are required to support the decisions. For example, investigators may conclude that there is no actionable link between food and clinical isolates, despite the determination that genome sequences are genetically identical or nearly identical. Also, links between isolates may exist, despite the presence of large numbers of SNPs between them. This approach is intended to reduce the chances that minor variations in a category of evidence or the lack of knowledge about the evolution of reservoir populations will lead to significant changes in the interpretation of WGS results.

**Table 2:** Conditions used to determine whether whole-genome sequence analyses support a match between two or more genomes.

	Supports	Neutral	Does not support
SNP distance	<21	21–100	>100
Bootstrap support	>0.89	0.80–0.89	<0.80
Tree topology	Monophyletic	Paraphyletic	Polyphyletic

The following examples of actual outbreaks and compliance cases investigated by the FDA illustrate the application of the interpretation framework (**Table 3**). These examples also show the different ways that the FDA uses WGS data during investigations.

**Table 3:** Characteristics of the examples presented in this paper.

Example	SNP distance	Bootstrap support	Tree topology	Epidemiology, traceback, or compliance findings	Conclusion
Identifying the source of an *E. coli* outbreak	Supports	Supports	Supports	Supports	Match
Matching food isolates from one firm to environmental isolates from another firm	Supports	Supports	Supports	Supports	Match
Identifying a resident pathogen	Supports	Supports	Supports	Not applicable	Not applicable
Populations of environmental isolates can be very diverse	Neutral	Supports	Supports	Not applicable	Not applicable
Analyzing paraphyletic relationships	Supports	Neutral	Neutral	Does not support	No match
Evidence that isolates arose from the same source by WGS does not necessarily mean that they are linked	Supports	Supports	Supports	Does not support	No match

## Examples

### Identifying the Source of an *E. coli* Outbreak

**FIGURE 3:**
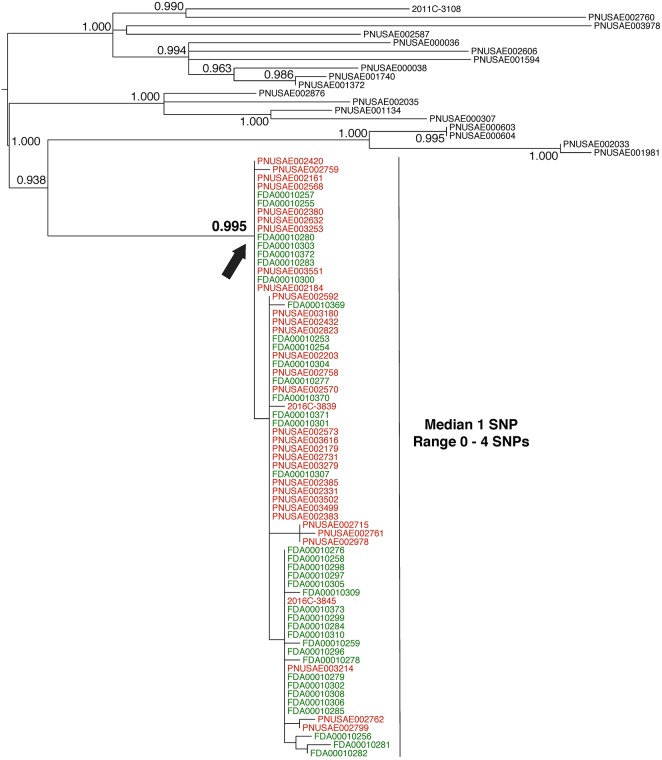
Phylogenetic analysis of genome sequences obtained from *Escherichia coli* isolates implicated in a 2016 flour outbreak. A SNP matrix was generated for 89 isolates with the CFSAN SNP pipeline v0.8.0 (Davis et al., 2015). The SNP matrix was phylogenetically analyzed with GARLI v2.01.1067 (Zwicki, 2006), using the K80 and HKY nucleotide substitution models. Branch labels indicate support values for 1000 bootstrap replicates. Bootstrap values less than 0.800 are not shown. Red labels indicate clinical isolates and green labels indicate flour isolates related to the outbreak. Black labels indicate isolates that were determined by epidemiology and traceback investigations to not be involved in the outbreak. The black arrow identifies the node that represents the last common ancestor of isolates included in the 2016 outbreak.

The first example is a straightforward case. The isolates are closely related, the evolutionary relationships are clear, and the epidemiological data support the relationships inferred by the phylogenetic analysis (**Figure 3**). From December 2015 to September 2016, there were 63 reported illnesses from Shiga toxin-producing *E. coli* in 24 states (Crowe et al., 2017). Epidemiologists discovered that patients had contact with raw flour before the onset of illness. Traceback investigations identified a flour producer as the possible source. *Escherichia coli* O121 was isolated from open packages of flour that were obtained from the residences of sickened people. GT laboratories sequenced these isolates.

Whole-genome sequence analysis of 37 flour (**Figure 3**, green labels) and 34 clinical isolates (**Figure 3**, red labels) indicates a median pairwise SNP distance of 1 (range 0–4). This value is well within the range of distances that are observed by researchers between isolates from the same source (**Table 1**). Also, the phylogenetic analysis supports the grouping of the food and clinical isolates to the exclusion of all other isolates in the GT database (i.e., they form a monophyletic group). The node on the tree that represents the common ancestor of the flour and clinical isolates (**Figure 3**, black arrow) has a strong bootstrap support value of 0.995 (meaning that the food and clinical isolates are monophyletic in 995 of 1000 replicate trees). All three types of WGS evidence support the hypothesis that the flour and clinical isolates originated from the same source. Furthermore, traceback investigations and epidemiological information confirm that contaminated flour from a specific firm was the vehicle for transmission of *E. coli* to consumers.

### Matching Food Isolates From One Firm to Environmental Isolates From Another Firm

In 2016, an ice cream firm (Producer) detected *L. monocytogenes* in new finished products. This firm had a prior history of *L. monocytogenes* contamination in both the food-processing environment and products. Due diligence by the firm, in cooperation with the FDA, revealed that the source was likely an ingredient provided by another firm (Supplier). The FDA collected samples from finished products from the Producer and environmental samples from the Supplier facility. *Listeria monocytogenes* was isolated from both sets of samples. We confirmed that the *L. monocytogenes* in the ingredient arose from a reservoir population in the Supplier’s facility, rather than in the Producer’s facility.

**FIGURE 4:**
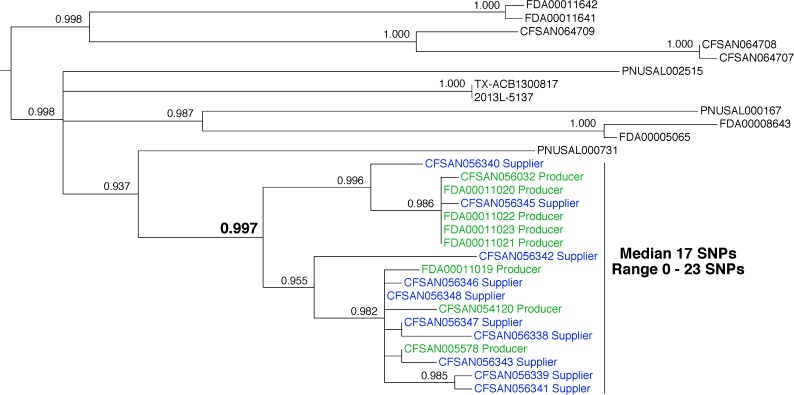
Phylogenetic analysis of genome sequences obtained from *Listeria monocytogenes* isolated from 2016 ice cream samples and the environment of a supplier. A SNP matrix was generated for 30 isolates with the CFSAN SNP pipeline v0.8.0 (Davis et al., 2015). The SNP matrix was phylogenetically analyzed with GARLI v2.01.1067 (Zwicki, 2006), using the K80 and HKY nucleotide substitution models. Branch labels indicate support values for 1000 bootstrap replicates. Bootstrap values less than 0.800 are not shown. Green labels indicate food isolates and blue labels indicate environmental isolates. Black labels indicate bacteria that were not isolated from either the ice cream samples or the supplier environment but are closely related.

We measured a median pairwise distance of 17 SNPs (range 0–23) between 8 *L. monocytogenes* that were isolated from food samples that were collected from Producer (**Figure 4**, green labels) and 10 environmental samples that were collected from the Supplier facility (**Figure 4**, blue labels). These distances suggest that the isolates are representatives of a genetically diverse reservoir population. Phylogenetic analysis shows that the food isolates from Producer and the environmental isolates from Supplier form a monophyletic group. The bootstrap analysis supports this relationship (0.997). Traceback investigations confirmed that the Producer used the ingredients from the Supplier in the production of ice cream products. The best interpretation of these data is that tainted ingredients provided by the Supplier led to contamination of the final ready-to-eat foods generated by the Producer.

This example demonstrates that WGS data can be useful in identifying ingredients as sources of contamination and shows how the FDA uses WGS to empower firms to identify sources of foodborne pathogens quickly and efficiently. This case also illustrates that populations of bacteria originating from a single food production facility can be diverse, making strict cutoff values for SNP distances inappropriate and consideration of other evidence, such as traceback evidence, imperative.

### Identifying a Resident Pathogen

**FIGURE 5:**
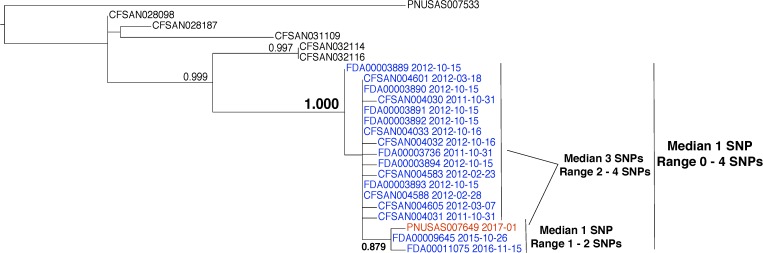
Phylogenetic analysis of genome sequences obtained from *Salmonella enterica* isolates collected from a food processing facility from 2011 to 2016. A SNP matrix was generated for 24 isolates with the CFSAN SNP pipeline v0.8.0 (Davis et al., 2015). The SNP matrix was phylogenetically analyzed with GARLI v2.01.1067 (Zwicki, 2006), using the K80 and HKY nucleotide substitution models. Branch labels indicate support values for 1000 bootstrap replicates. Bootstrap values less than 0.800 are not shown. Blue labels indicate environmental isolates and the red label indicates a clinical isolate. Black labels indicate isolates that did not originate from the subject food processing facility but are closely related.

This example indicates the presence of a resident pathogen within a facility. *Salmonella enterica* was isolated from environmental samples that were collected from the same facility during inspections that occurred in 2011, 2012, 2015, and 2016 (**Figure 5**, blue labels). Pairwise distance analysis indicates a median of 1 SNP (range 0–4). Phylogenetic analysis reveals that they are monophyletic and that there is strong bootstrap support for this relationship (1.000). Interestingly, the isolates that were collected during the 2015 and 2016 inspections form a group that is median 3 SNPs distant (range 2–4) from the sequences that were collected during the 2011 and 2012 inspections. This could indicate that representatives of different subpopulations were isolated. It may also indicate that the population evolved at a rate of approximately 0.75 nucleotides per year. This rate is slower than the laboratory measurement of 4.70 nucleotides per year (Duchene et al., 2016). However, it is consistent with a study of foodborne *S. enterica*, during which an evolutionary rate of 1.01 nucleotide substitutions per year was measured (Deng et al., 2014). These differences are likely due to variations in selection, generation times, and population sizes (Duchene et al., 2016). The bootstrap support for the separate grouping of isolates collected in 2015 and 2016 is in the neutral range (0.879). We expect lower bootstrap values when small numbers of SNPs separate clusters of isolates. There is no reason to expect this same evolutionary rate should apply to all food-processing environments. Isolates in different environments will experience different evolutionary events. However, this example demonstrates that nucleotide changes likely occur within populations of resident pathogens.

The environmental isolates collected in 2015 and 2016 are closely related to a clinical isolate that was collected in 2017 (**Figure 5**, red label). The bootstrap support for the grouping of the clinical isolate with the 2015 and 2016 environmental isolates is in the neutral range (0.879). However, there is strong support for its grouping with the entire set of environmental isolates that were collected from this firm (1.000). Furthermore, the clinical and environmental isolates group together to the exclusion of all other isolates in the GT database. This evidence supports a match between the environmental and clinical isolates. But, support for a match between the 2015 and 2016 environmental isolates and the clinical isolate is moderate and the relationship between them is less clear. This result implies that the environmental and clinical isolates arose from the same source. However, investigators did not discover an epidemiological link between them.

### Populations of Environmental Isolates Can Be Very Diverse

**FIGURE 6:**
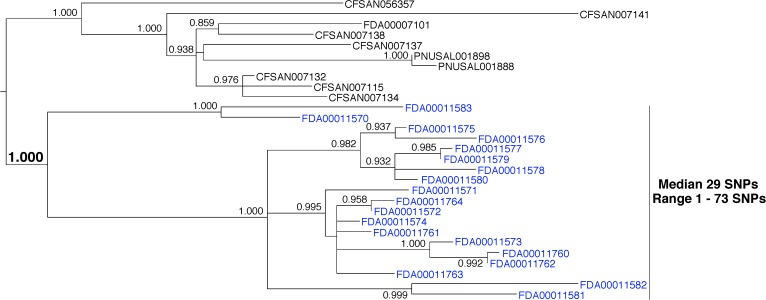
Phylogenetic analysis of genome sequences obtained from *Listeria monocytogenes* isolates collected from a food processing facility during a single inspection. A SNP matrix was generated for 29 isolates with the CFSAN SNP pipeline v0.8.0 (Davis et al., 2015). The SNP matrix was phylogenetically analyzed with GARLI v2.01.1067 (Zwicki, 2006), using the K80 and HKY nucleotide substitution models. Branch labels indicate support values for 1000 bootstrap replicates. Bootstrap values less than 0.800 are not shown. Blue labels indicate environmental isolates. Black labels indicate isolates that did not originate from the subject food processing facility but are closely related.

Whole-genome sequence data from 19 *L. monocytogenes* environmental isolates that were collected during a single inspection were analyzed (**Figure 6**, blue labels). The analysis indicates a median pairwise distance of 29 SNPs (range 1–73). The phylogenetic analysis reveals that the isolates group together to the exclusion of all others in the database with strong bootstrap support (1.000). Although the pairwise distances measured here are high, they are consistent with at least one published study (Jackson et al., 2016). This example underscores the need for a sophisticated framework that supports matches in a variety of conditions, including the origination of isolates from very diverse reservoir populations.

### Analyzing Paraphyletic Relationships

**FIGURE 7:**
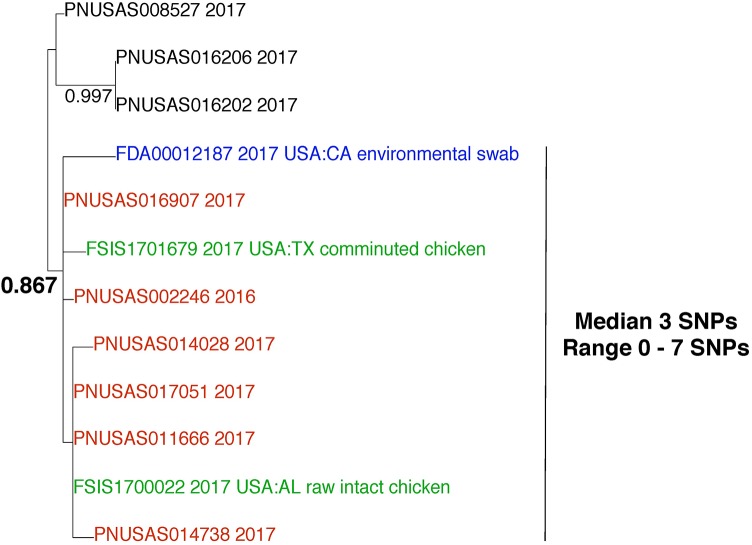
Phylogenetic analysis of genome sequences obtained from *Salmonella enterica* isolated from environmental, food, and clinical samples. A SNP matrix was generated for 12 isolates with the CFSAN SNP pipeline v0.8.0 (Davis et al., 2015). The SNP matrix was phylogenetically analyzed with GARLI v2.01.1067 (Zwicki, 2006), using the K80 and HKY nucleotide substitution models. Branch labels indicate support values for 1000 bootstrap replicates. Bootstrap values less than 0.800 are not shown. Blue labels indicate environmental isolates, red labels indicate clinical isolates, and green labels indicate food isolates. Black labels indicate isolates that are not the focus of this example but are closely related.

This example demonstrates the importance of including all data from the GT database in WGS analyses. *Salmonella enterica* was isolated from an environmental sample that was collected from a food processing facility. We queried the WGS data against the entire GT *S. enterica* database at NCBI. We included all closely related isolates in downstream analyses. Pairwise SNP analysis indicates that the environmental isolate (**Figure 7**; blue) is closely related to 6 clinical isolates (red) and 2 food isolates (green), with median 3 SNPs between them (range 0–7). This result supports the hypothesis that these isolates originated from the same source. The phylogenetic analysis reveals that these isolates group together. The bootstrap result is within the neutral range of support (0.867). If we consider the relationship of the environmental isolate (blue) to the clinical isolates (red), we can see that they group together but not to the exclusion of all other isolates. This evidence indicates that a paraphyletic relationship exists between these isolates. It is possible for investigators to establish a link between the environmental isolate and the clinical isolates with epidemiological and traceback evidence. Ideally, investigators should also explore the relationships of the environmental and clinical isolates to the food isolates. In this case, there was no epidemiological or traceback evidence to show that a link exists between the environmental and the clinical isolates.

### Evidence That Isolates Arose From the Same Source by WGS Does Not Necessarily Mean That They Are Linked

**FIGURE 8:**
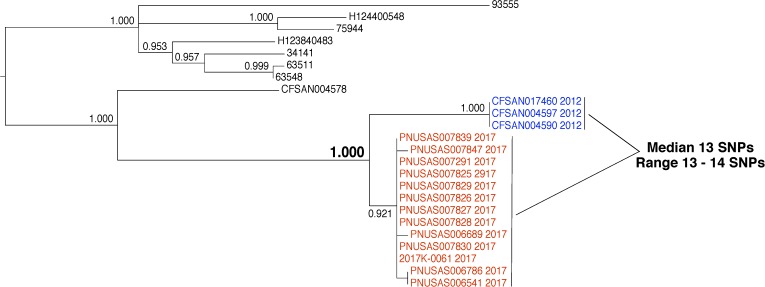
Phylogenetic analysis of genome sequences obtained from *Salmonella enterica* isolates collected from a food processing facility and closely related clinical isolates. A SNP matrix was generated for 24 isolates with the CFSAN SNP pipeline v0.8.0 (Davis et al., 2015). The SNP matrix was phylogenetically analyzed with GARLI v2.01.1067 (Zwicki, 2006), using the K80 and HKY nucleotide substitution models. Branch labels indicate support values for 1000 bootstrap replicates. Bootstrap values less than 0.800 are not shown. Blue labels indicate environmental isolates and red labels indicate clinical isolates. Black labels indicate isolates that are not the focus of this example but are closely related.

In 2012, three *S. enterica* were isolated from environmental samples that were collected from a food processing facility (**Figure 8**, blue labels). The results of WGS analysis indicate a close evolutionary relationship between the environmental isolates and 13 clinical isolates collected in 2017 (**Figure 8**, red labels). We measured a median genetic distance of 13 SNPs (range 13–14) between the environmental and clinical isolates. The isolates group together to the exclusion of all other isolates in the database with strong bootstrap support (1.000). This evidence indicates that these isolates arose from the same source. However, inspectors collected the environmental isolates in 2012 and the patients were sickened in 2017. Also, there was no epidemiological signal for the commodity produced by this facility. Finally, no traceback evidence linked the patients to the food product. Therefore, the connection between these environmental and clinical isolates is unclear. A conclusion that the isolates originated from the same source by WGS analysis can be made but the epidemiological and traceback evidence do not link them. The tree topology offers more information that suggests the isolates might not be linked. The environmental and clinical isolates form distinct subgroups, rather than being interspersed. These data show that although the clinical isolates originated from the same source of contamination, that source may not have been the food processing facility. With SNP thresholds, it is possible that investigators could have erroneously concluded that product from the food processing facility caused the illnesses.

## Challenges and Limitations

Whole-genome sequence analysis is a powerful tool for determining the relatedness of bacterial isolates. However, it can only indicate that isolates recently arose from the same source. Epidemiology and traceback evidence are still needed to infer the location of that source and to link isolates. A determination that clinical isolates originated from the same source as food or environmental isolates alone should not lead to the presumption that the contaminated food or environment caused illness. This limitation is significant as causal links between isolates can have substantial consequences for firms.

Another limitation is the dependence of analyses on genome sequence databases. Although the GT database of foodborne pathogens is the largest and most complete of its kind, there are potential biases in what data laboratories collect and deposit. Submission of WGS data that was obtained from clinical samples is limited to only those people that sought medical care for their illnesses. Furthermore, patients must have received care at facilities that are equipped to take samples from which bacteria may be isolated. Environmental isolates that are included in the database are limited to those food-processing facilities that yield positive samples. The result is that the GT database does not represent the full genetic diversities of the bacterial populations that exist within facilities. Similarly, food isolates in the database are limited to those commodities that test positive for foodborne pathogens. Such biased sampling is, of course, a result of limited resources and the regulatory framework within which the tracking of foodborne pathogens is done. These realities must be kept in mind when interpreting the results of WGS analyses in the context of food safety.

## Conclusion

Whole-genome sequence analysis of foodborne bacterial pathogens is transforming source attribution studies. However, a full understanding of the correct interpretation of WGS analyses is a work in progress. Analysis of bacterial pathogens during illness outbreaks or regulatory activities involves the comparison of one representative of a changing population of organisms to another representative of a changing population with the goal of determining whether they originated from the same source. This application of DNA sequence data is a study of the population genetics of foodborne pathogens. The use of DNA sequence data in the context of food safety, then, presents challenges to the interpretation of WGS results. These challenges should be kept in mind as investigators interpret WGS results and communicate them to industry stakeholders and the public.

Often, there is an expectation that genome sequences need to be identical (or nearly identical) for matches to be made. However, the existence of large numbers of SNPs between genomes does not exclude the possibility of a match by WGS analysis. Differences between isolates are expected. Thus, guidance on the criteria that define a match needs to incorporate the occurrences of unknown numbers of significant evolutionary events.

The goal of this paper is to clarify the thinking behind our interpretation of WGS data and to promote transparency. WGS analyses should be interpreted in conjunction with epidemiology and traceback findings. Conclusions should be based upon a preponderance of the evidence. This framework should not be adhered to blindly or rigidly. Also, it should be reassessed as the food safety community learns more about WGS analysis and as other groups publish their guidelines. Interpreting WGS results requires significant flexibility to incorporate all relevant information, with the implications of the findings taken into account, and with the aim of ensuring a safe food supply.

## Data Availability

The datasets analyzed for this study are stored in the National Center for Biotechnology Information’s GenomeTrakr sequence repository (ncbi.nlm.nih.gov/bioproject/?term=genometrakr).

## Author Contributions

AP, JP, YL, JB, HR, and ES conceived the project, developed the framework, and drafted the paper. AP, JP, YL, and JB analyzed the sequence data.

## Conflict of Interest Statement

The authors declare that the research was conducted in the absence of any commercial or financial relationships that could be construed as a potential conflict of interest.

## Footnotes

1 https://www.fda.gov/food/foodscienceresearch/wholegenomesequencingprogramwgs/ucm363134.htm

2 ncbi.nlm.nih.gov/pathogens

3 ncbi.nlm.nih.gov/pathogens/isolates

4 ncbi.nlm.nih.gov/bioproject/?term=genometrakr

5 ftp.ncbi.nlm.nih.gov/pathogen/Methods.txt

## References

[B1] Allard M. W. Luo Y. Strain E. Li C. Keys C. E. Son I. (2012). High resolution clustering of *Salmonella enterica* serovar Montevideo strains using a next-generation sequencing approach. *BMC Genomics* 13:32. 10.1186/1471-2164-13-32 22260654 PMC3368722

[B2] Allard M. W. Strain E. Melka D. Bunning K. Musser S. M. Brown E. W. (2016). Practical value of food pathogen traceability through building a whole-genome sequencing network and database. *J. Clin. Microbiol.* 54 1975–1983. 10.1128/JCM.00081-16 27008877 PMC4963501

[B3] Aquilina G. Azimonti G. Bampidis V. de Lourdes Bastos M. Bories G. Chesson A. (2018). Guidance on the characterisation of microorganisms used as feed additives or as production organisms. *EFSA J.* 16:5206. 10.2903/j.efsa.2018.5206 PMC7009341 32625840

[B4] Chen Y. Burall L. S. Luo Y. Timme R. Melka D. Muruvanda T. (2016). *Listeria monocytogenes* in stone fruits linked to a multistate outbreak: enumeration of cells and whole-genome sequencing. *Appl. Environ. Microbiol.* 82 7030–7040. 10.1128/AEM.01486-1416 27694232 PMC5118914

[B5] Chen Y. Luo Y. Carleton H. Timme R. Melka D. Muruvanda T. (2017a). Whole genome and core genome multilocus sequence typing and single nucleotide polymorphism analyses of *Listeria monocytogenes* associated with an outbreak linked to cheese, United States, 2013. *Appl. Environ. Microbiol.* 10.1128/AEM.00633-617 [Epub ahead of print]. 28550058 PMC5514676

[B6] Chen Y. Luo Y. Curry P. Timme R. Melka D. Doyle M. (2017b). Assessing the genome level diversity of *Listeria monocytogenes* from contaminated ice cream and environmental samples linked to a *Listeriosis* outbreak in the United States. *PLoS One* 12:e0171389. 10.1371/journal.pone.0171389 28166293 PMC5293252

[B7] Chen Y. Luo Y. Pettengill J. Timme R. Melka D. Doyle M. (2017c). Singleton sequence type 382, an emerging clonal group of *Listeria monocytogenes* associated with three multistate outbreaks linked to contaminated stone fruit, caramel apples, and leafy green salad. *J. Clin. Microbiol.* 55 931–941. 10.1128/JCM.02140-2116 28053218 PMC5328462

[B8] Crowe S. J. Bottichio L. Shade L. N. Whitney B. M. Corral N. Melius B. (2017). Shiga toxin-producing *E. coli* infections associated with flour. *N. Engl. J. Med.* 377 2036–2043. 10.1056/NEJMoa1615910 29166238 PMC5792826

[B9] Davis S. Pettengill J. B. Luo Y. Payne J. Shpuntoff A. Rand H. (2015). CFSAN SNP pipeline: an automated method for constructing SNP matrices from next-generation sequence data. *Peerj Comp. Sci.* 1:e20 10.7717/peerj-cs.20

[B10] den Bakker H. C. Allard M. W. Bopp D. Brown E. W. Fontana J. Iqbal Z. (2014). Rapid whole-genome sequencing for surveillance of *Salmonella enterica* serovar Enteritidis. *Emerg. Infect. Dis.* 20 1306–1314. 10.3201/eid2008.131399 25062035 PMC4111163

[B11] Deng X. Desai P. T. den Bakker H. C. Mikoleit M. Tolar B. Trees E. (2014). Genomic epidemiology of *Salmonella enterica* serotype Enteritidis based on population structure of prevalent lineages. *Emerg. Infect. Dis.* 20 1481–1489. 10.3201/eid2009.131095 25147968 PMC4178404

[B12] Duchene S. Holt K. E. Weill F. X. Le Hello S. Hawkey J. Edwards D. J. (2016). Genome-scale rates of evolutionary change in bacteria. *Microb. Genom.* 2:e000094. 10.1099/mgen.0.000094 28348834 PMC5320706

[B13] Eppinger M. Mammel M. K. Leclerc J. E. Ravel J. Cebula T. A. (2011). Genomic anatomy of *Escherichia coli* O157:H7 outbreaks. *Proc. Natl. Acad. Sci. U.S.A.* 108 20142–20147. 10.1073/pnas.1107176108 22135463 PMC3250189

[B14] Farber J. M. (1996). An introduction to the hows and whys of molecular typing. *J. Food Prot.* 59 1091–1101. 10.4315/0362-028X-59.10.1091 31195469

[B15] Felsenstein J. (1985). Confidence limits on phylogenies: an approach using the bootstrap. *Evolution* 39 783–791. 10.1111/j.1558-5646.1985.tb00420.x 28561359

[B16] Ferreira V. Wiedmann M. Teixeira P. Stasiewicz M. J. (2014). *Listeria monocytogenes* persistence in food-associated environments: epidemiology, strain characteristics, and implications for public health. *J. Food. Prot.* 77 150–170. 10.4315/0362-028X.JFP-13-150 24406014

[B17] Gilmour M. W. Graham M. Van Domselaar G. Tyler S. Kent H. Trout-Yakel K. M. (2010). High-throughput genome sequencing of two *Listeria monocytogenes* clinical isolates during a large foodborne outbreak. *BMC Genomics* 11:120. 10.1186/1471-2164-11-120 20167121 PMC2834635

[B18] Griffiths E. Dooley D. Graham M. Van Domselaar G. Brinkman F. S. L. Hsiao W. W. L. (2017). Context is everything: harmonization of critical food microbiology descriptors and metadata for improved food safety and surveillance. *Front. Microbiol.* 8:1068. 10.3389/fmicb.2017.01068 28694792 PMC5483436

[B19] Grocery Manufacturers Association (2011). *Capturing Recall Costs: Measuring and Recovering the Losses [Online]. Grocery Manufacters Association.* Available at: gmaonline.org/forms/store/ProductFormPublic/capturing-recall-costs (Accessed April 9, 2018).

[B20] Hoffman S. Maculloch B. Batz M. (2015). *Economic Burden of Major Foodborne Illnesses Acquired in the United States [Online]. United States Department of Agriculture.* Available at: ers.usda.gov/webdocs/publications/43984/52806_eib140_summary.pdf?v = 42136 (accessed April 9, 2018).

[B21] Hoffmann M. Luo Y. Monday S. R. Gonzalez-Escalona N. Ottesen A. R. Muruvanda T. (2016). Tracing origins of the *Salmonella* Bareilly strain causing a food-borne outbreak in the United States. *J. Infect. Dis.* 213 502–508. 10.1093/infdis/jiv297 25995194

[B22] Imanishi M. Rotstein D. S. Reimschuessel R. Schwensohn C. A. Woody D. H. Jr. (2014). Outbreak of *Salmonella enterica* serotype infantis infection in humans linked to dry dog food in the United States and Canada, 2012. *J. Am. Vet. Med. Assoc.* 244 545–553. 10.2460/javma.244.5.545 24548229 PMC11292585

[B23] Jackson B. R. Tarr C. Strain E. Jackson K. A. Conrad A. Carleton H. (2016). Implementation of nationwide real-time whole-genome sequencing to enhance listeriosis outbreak detection and investigation. *Clin. Infect. Dis.* 63 380–386. 10.1093/cid/ciw242 27090985 PMC4946012

[B24] Keener L. Nicholson-Keener S. M. Koutchma T. (2014). Harmonization of legislation and regulations to achieve food safety: US and Canada perspective. *J. Sci. Food Agric.* 94 1947–1953. 10.1002/jsfa.6295 23818402

[B25] Kruse H. (1999). Globalization of the food supply-food safety implications: special regional requirements: future concerns. *Food Control* 10 315–320. 10.1016/S0956-7135(99)00005-5

[B26] Lambert D. Pightling A. Griffiths E. Van Domselaar G. Evans P. Berthelet S. (2017). Baseline practices for the application of genomic data supporting regulatory food safety. *J. AOAC Int.* 100 721–731. 10.5740/jaoacint.16-0269 28105974

[B27] Leekitcharoenphon P. Nielsen E. M. Kaas R. S. Lund O. Aarestrup F. M. (2014). Evaluation of whole genome sequencing for outbreak detection of *Salmonella enterica*. *PLoS One* 9:e87991. 10.1371/journal.pone.0087991 24505344 PMC3913712

[B28] Li Z. Perez-Osorio A. Wang Y. Eckmann K. Glover W. A. Allard M. W. (2017). Whole genome sequencing analyses of *Listeria monocytogenes* that persisted in a milkshake machine for a year and caused illnesses in washington state. *BMC Microbiol.* 17:134. 10.1186/s12866-017-1043-1041 28619007 PMC5472956

[B29] Moran-Gilad J. Sintchenko V. Pedersen S. K. Wolfgang W. J. Pettengill J. Strain E. (2015). Proficiency testing for bacterial whole genome sequencing: an end-user survey of current capabilities, requirements and priorities. *BMC Infect. Dis.* 15:174. 10.1186/s12879-015-0902-903 25887164 PMC4392855

[B30] Moura A. Criscuolo A. Pouseele H. Maury M. M. Leclercq A. Tarr C. (2016). Whole genome-based population biology and epidemiological surveillance of *Listeria monocytogenes*. *Nat. Microbiol.* 2:16185. 10.1038/nmicrobiol.2016.185 27723724 PMC8903085

[B31] Nielsen E. M. Bjorkman J. T. Kiil K. Grant K. Dallman T. Painset A. (2017). *Closing Gaps for Performing a Risk Assessment on Listeria monocytogenes in Ready-To-Eat (RTE) Foods: Activity 3 the Comparison of Isolates from Different Compartments along the Food Chain, and from Humans Using Whole Genome Sequencing (WGS) Analysis.* Parma: European Food Safety Authority.

[B32] Octavia S. Wang Q. Tanaka M. M. Kaur S. Sintchenko V. Lan R. (2015). Delineating community outbreaks of *Salmonella enterica* serovar typhimurium by use of whole-genome sequencing: insights into genomic variability within an outbreak. *J. Clin. Microbiol.* 53 1063–1071. 10.1128/JCM.03235-3214 25609719 PMC4365247

[B33] Pightling A. W. Petronella N. Pagotto F. (2014). Choice of reference sequence and assembler for alignment of *Listeria monocytogenes* short-read sequence data greatly influences rates of error in SNP analyses. *PLoS One* 9:e104579. 10.1371/journal.pone.0104579 25144537 PMC4140716

[B34] Pightling A. W. Petronella N. Pagotto F. (2015). The *Listeria monocytogenes* core-genome sequence typer (LmCGST): a bioinformatic pipeline for molecular characterization with next-generation sequence data. *BMC Microbiol.* 15:224. 10.1186/s12866-015-0526-521 26490433 PMC4618880

[B35] Self J. L. Conrad A. Stroika S. Jackson A. Burnworth L. Beal J. (2016). Notes from the field: outbreak of *Listeriosis* associated with consumption of package salad – United States and Canada, 2015-2016. *MMWR Morb. Mortal. Wkly. Rep.* 879–881. 10.15585/mmwr.mm6533a6 27559935

[B36] Snitkin E. S. Zelazny A. M. Thomas P. J. Stock F. NISC Comparative Sequencing Program Group Henderson D. K., (2012). Tracking a hospital outbreak of carbapenem-resistant *Klebsiella pneumoniae* with whole-genome sequencing. *Sci. Transl. Med.* 4:148ra116. 10.1126/scitranslmed.3004129 22914622 PMC3521604

[B37] Spratt B. G. (2004). Exploring the concept of clonality in bacteria. *Methods Mol. Biol.* 266 323–352. 10.1385/1-59259-763-7:323 15148426

[B38] Stasiewicz M. J. Oliver H. F. Wiedmann M. den Bakker H. C. (2015). Whole-genome sequencing allows for improved identification of persistent *Listeria monocytogenes* in food-associated environments. *Appl. Environ. Microbiol.* 81 6024–6037. 10.1128/AEM.01049-1015 26116683 PMC4551262

[B39] Stevens E. L. Timme R. Brown E. W. Allard M. W. Strain E. Bunning K. (2017). The public health impact of a publically available, environmental database of microbial genomes. *Front. Microbiol.* 8:808. 10.3389/fmicb.2017.00808 28536563 PMC5422427

[B40] Taylor A. J. Lappi V. Wolfgang W. J. Lapierre P. Palumbo M. J. Medus C. (2015). Characterization of foodborne outbreaks of *Salmonella enterica* serovar Enteritidis with whole-genome sequencing single nucleotide polymorphism-based analysis for surveillance and outbreak detection. *J. Clin. Microbiol.* 53 3334–3340. 10.1128/JCM.01280-1215 26269623 PMC4572550

[B41] Underwood A. P. Dallman T. Thomson N. R. Williams M. Harker K. Perry N. (2013). Public health value of next-generation DNA sequencing of enterohemorrhagic *Escherichia coli* isolates from an outbreak. *J. Clin. Microbiol.* 51 232–237. 10.1128/JCM.01696-1612 23135946 PMC3536255

[B42] Wang Q. Holmes N. Martinez E. Howard P. Hill-Cawthorne G. Sintchenko V. (2015). It is not all about single nucleotide polymorphisms: comparison of mobile genetic elements and deletions in *Listeria monocytogenes* genomes links cases of hospital-acquired *Listeriosis* to the environmental source. *J. Clin. Microbiol.* 53 3492–3500. 10.1128/JCM.00202-215 26311854 PMC4609684

[B43] Wilson M. R. Allard M. W. Brown E. W. (2013). The forensic analysis of foodborne bacterial pathogens in the age of whole-genome sequencing. *Cladistics* 29 449–461. 10.1111/cla.12012 34798769

[B44] Wuyts V. Denayer S. Roosens N. H. C. Mattheus W. Bertrand S. Marchal K. (2015). Whole genome sequence analysis of *Salmonella* Enteritidis PT4 outbreaks from a national reference laboratory’s viewpoint. *PLoS Curr. Outbreaks.* 10.1371/currents.outbreaks.aa5372d90826e6cb0136ff66bb7a62fc 26468422 PMC4593640

[B45] Zwicki D. J. (2006). *Genetic Algorithm Approaches for the Phylogenetic Analysis of Large Biological Sequence Datasets Under the Maximum Likelihood Criterion.* Ph.D. dissertation, The University of Texas at Austin, Austin, TX.

